# Soluble guanylyl cyclase: Molecular basis for ligand selectivity and action *in vitro* and *in vivo*

**DOI:** 10.3389/fmolb.2022.1007768

**Published:** 2022-10-11

**Authors:** Gang Wu, Iraida Sharina, Emil Martin

**Affiliations:** ^1^ Hematology-Oncology Division, Department of Internal Medicine, The University of Texas—McGovern Medical School, Houston, TX, United States; ^2^ Cardiology Division, Department of Internal Medicine, The University of Texas—McGovern Medical School, Houston, TX, United States

**Keywords:** nitric oxide, hemoprotein, soluble guanylyl cyclase, cGMP, ligand selectivity

## Abstract

Nitric oxide (NO), carbon monoxide (CO), oxygen (O_2_), hydrogen sulfide (H_2_S) are gaseous molecules that play important roles in the physiology and pathophysiology of eukaryotes. Tissue concentrations of these physiologically relevant gases vary remarkable from nM range for NO to high μM range of O_2_. Various hemoproteins play a significant role in sensing and transducing cellular signals encoded by gaseous molecules or in transporting them. Soluble guanylyl cyclase (sGC) is a hemoprotein that plays vital roles in a wide range of physiological functions and combines the functions of gaseous sensor and signal transducer. sGC uniquely evolved to sense low non-toxic levels of NO and respond to elevated NO levels by increasing its catalytic ability to generate the secondary signaling messenger cyclic guanosine monophosphate (cGMP). This review discusses sGC’s gaseous ligand selectivity and the molecular basis for sGC function as high-affinity and selectivity NO receptor. The effects of other gaseous molecules and small molecules of cellular origin on sGC’s function are also discussed.

## 1 Introduction

### 1.1 Soluble guanylyl cyclase–A highly specific receptor for nitric oxide

Carbon monoxide (CO), nitric oxide (NO), oxygen (O_2_) and hydrogen sulfide (H_2_S) are gas molecules that are similar not only due to their small size, similar water solubility and membrane permeability but also the fact that they can be generated by different living organisms. In many cases these gaseous molecules are essential for cellular communications and act as signaling molecules that trigger various physiological responses (Girvan and Munro, 2013; Liebl et al., 2013). The concentrations of these gaseous signaling molecules widely fluctuate, depending on the tissue in question or physiological status. For example, endogenous CO concentration was estimated at low nM levels (Coburn et al., 1963; Coburn, 1973; Marks et al., 2002), NO concentrations were recorded from nM to µM range (Lundberg et al., 2008), H_2_S is found at sub-micromolar level (Stein and Bailey, 2013), while oxygen concentration can reach hundreds of µM in oxygenated tissues.

Living organisms have evolved various proteins to sense these gaseous messengers. Heme-containing proteins play a central role in this process and are often referred to as heme-based gas sensors (Shimizu et al., 2015). Many heme-based sensors evolved to be highly selective and sensitive to the effect of only one of these gases. Soluble guanylyl cyclase (sGC) with an enzymatic function of cGMP synthesis from GTP is a recognized mammalian receptor for NO and is currently classified as NO-sensitive guanylyl cyclase (NO-GC). In this review, we will use the traditional nomenclature and refer to the enzyme as sGC. The NO sensing capability of sGC is conferred by a ferrous heme moiety. Binding of NO to this heme moiety results in several hundred fold increase in cGMP-forming activity of sGC. Thus, sGC combines the ability to sense transient fluxes of cellular NO with the ability to increase intracellular cGMP level. Increase of intracellular cGMP synthesis results in activation of cGMP effector molecules, such as cGMP-regulated phosphodiesterases, cGMP-dependent protein kinases and cyclic nucleotide-gated ion channels. Thus, sGC plays the important role of NO-induced elevation of cGMP level, leading to a variety of cellular and physiological events. The effects of sGC activation include calcium sequestration and cytoskeletal changes, relaxation of vascular smooth muscle cells (VSMC) and improved oxygenation of tissues and organs (Stasch et al., 2001), facilitation of the repair of injured endothelium (Noiri et al., 1997; Noiri et al., 1998), inhibition of adhesion and subsequent migration of leukocytes (Kubes et al., 1991), reduction of platelet aggregation (Radomski et al., 1987; Makhoul et al., 2018), and inhibition of proliferation and migration of VSMCs (Seki et al., 1995), regulation of gastrointestinal motility (Friebe et al., 2007), modulation of cancer development (Zhu et al., 2011). It is not surprising that sGC is an important and active therapeutic target. NO-generating nitrovasodilators such as nitroglycerin and isosorbide mononitrate are used to upregulate sGC function to manage heart failure, angina, and hypertensive crisis (Boerrigter et al., 2009). Understanding the molecular events that govern the function of sGC as NO receptor has important clinical applications. More recently, a number of NO-independent regulators of NO-GC have been identified and even approved for clinical use (Sandner et al., 2019).

sGC exhibits an extremely high affinity for NO, but excludes any binding of O_2_, and is thus capable of sensing less than nM [NO] under aerobic conditions, where [O_2_] is ∼260 µM. In this review, we will analyze the process of NO binding to sGC heme and discuss the factors that determine the gaseous ligand discrimination by sGC in the context of simple homogeneous *in vitro* system and more complex heterogeneous intracellular conditions.

### 1.2 Structure of Soluble guanylyl cyclase

**FIGURE 1:**
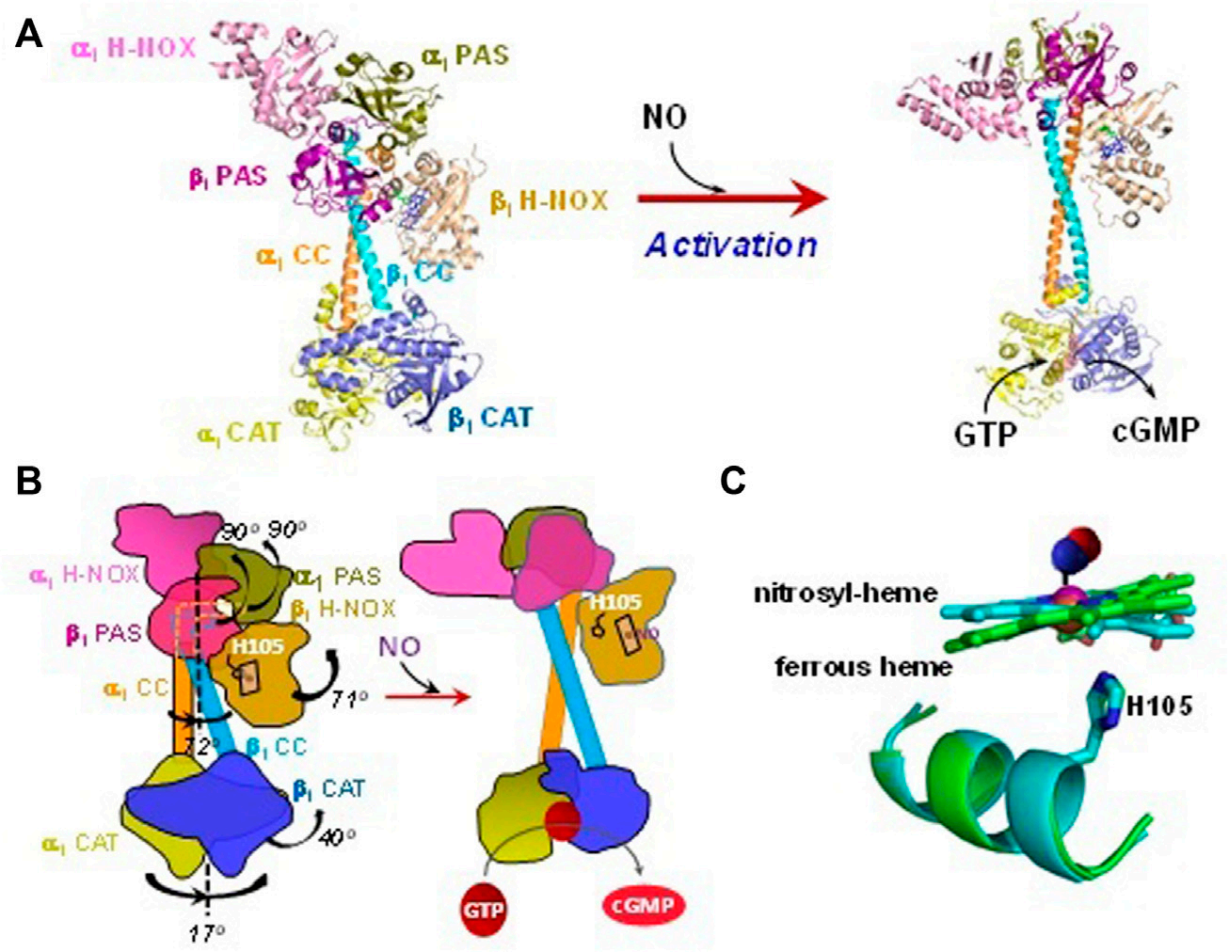
Structural changes of sGC in response to NO-dependent activation. **(A)** The structures in different states are plotted using PyMol: Unliganded ferrous state (PDB 6JT0) and NO-bound activated state (PDB 6JT2). **(B)** Cartoons to demonstrate structural rearrangement of the domains in sGC upon NO-activation. **(C)** Heme and the proximal ligand His105 are overlapped to show the conformational change in resting and NO bound states. The NO molecule, which is not visible in structure PDB 6JT2, is manually added in a random configuration.

Soluble GC is a heterodimer, consisting of *α* and *β* subunits. Humans and mice have two functional isoforms of the *α* subunit (*α*_1_ and *α*_2_) and one functional *ß*_1_ isoform. The heterodimer α_1_β_1,_ now classified as GC-1, is ubiquitously expressed and has a higher level of expression than the α_2_β_1_ (GC-2) counterpart, which has tissue-specific expression, more prevalent in brain, kidney and placenta. In this review, we focus primarily on the properties of GC-1 isoform, which is much better studied. While there might be some minor differences between the affinities to gaseous ligands, the association and dissociation rates, the overall strategy of ligand discrimination should be similar for both GC-1 and GC-2 isoforms. Each sGC subunit contains a heme nitric oxide/oxygen binding domain (H-NOX), a Per-Arnt-Sim domain (PAS), a coiled-coil domain (CC) and a catalytic domain (CAT). Only the *ß*_1_ H-NOX domain contains a heme prosthetic group (Derbyshire and Marletta, 2012). Cryogenic electron microscopy (cryo-EM) of dimeric sGC shows a two-lope structure with the H-NOX/PAS domains on one end and the CAT domains on the other, connected by the CC domains. The structure reveals that *ß*_1_ CC helix has a contact with the *ß*_1_ H-NOX domain and the CC domains form a bend in resting sGC (Figures 1A,B). In this state the enzyme has a low cGMP-forming activity and is often referred to as a low cGMP output form. Soluble GC isolated from native and heterologous expression sources contains a pentacoordinate high-spin ferrous heme (5c sGC) with histidine 105 (His105) as the proximal ligand (Stone and Marletta, 1994; Martin et al., 2005a). NO binding to the heme causes the cleavage of the heme-His105 coordinate bond (Figure 1C). This in turn triggers a relative rotation among *α*_1_ and *ß*_1_ CC helices and straightening of the CC domains, while some interactions between the *ß*_1_ CC helix and the heme-containing *ß*_1_ H-NOX domain are retained (Figures 1A,B). Transduction of the CC rotation to the CAT domains leads to rotational conformational changes in the latter, resulting in optimization of the GTP substrate binding pocket (Horst and Marletta, 2018; Kang et al., 2019). While these conformational changes only marginally improve the K_M_ for GTP, the V_max_ is increased several hundred times (Lee et al., 2000). In this activated state sGC is considered to be in a high cGMP output state with turnover number of over 3,000/min (Martin et al., 2005a).

### 1.3 Gaseous ligand selectivity of sGC

#### 1.3.1 Effect of hydrogen sulfide on sGC activity and function

Hydrogen sulfide (H_2_S) has emerged as a new endogenous gasotransmitter that is involved in a broad range of physiological functions. Three enzymes are recognized as endogenous sources of H_2_S in various cells and tissues: cystathionine γ-lyase (CSE), cystathionine β-synthase (CBS), and 3-mercaptopyruvate sulfurtransferase (Cirino et al., 2022). The wide range of physiological processes affected by H_2_S has a significant overlap with the diverse physiological effects of NO. Moreover, studies demonstrate that NO and H_2_S signaling are mutually dependent in the regulation of angiogenesis and endothelium-dependent relaxation (Coletta et al., 2012). The primary recognized mechanism through which H_2_S affects the activity of signaling proteins is persulfidation of reactive cysteine residues on target proteins with the formation of persulfide group (-SSH) (Paul and Snyder, 2015). Soluble GC has a large number of conserved cysteines that could potentially be a target of redox modification (Beuve, 2017), including persulfidation. However, when purified sGC was directly exposed to H_2_S donor, no change in cGMP-forming activity of sGC was observed (Coletta et al., 2012). The role of H_2_S gasotransmitter in the function of sGC was revealed when sGC with oxidized heme was exposed to H_2_S. Soluble GC containing oxidized ferric heme does not bind NO and is not subject to NO activation. However, following the treatment with H_2_S, sGC with ferric heme restored its ability to bind NO and stimulated cGMP-forming activity (Zhou et al., 2016). UV-Vis spectral analysis of sGC with ferric heme revealed that in the presence of H_2_S an efficient reduction of the heme iron and restoration of a fully functional and NO-responsive ferrous heme sGC takes place. At the same time, no evidence of H_2_S-dependent sulfheme modification of the porphyrin ring was observed. Studies in cultured rat aortic smooth muscle cells and mouse aortic rings provided evidence that H_2_S-mediated reduction of ferric sGC heme happens not only in homogeneous purified systems, but it is a physiological process that facilitates NO-mediated cellular signaling events (Zhou et al., 2016).

#### 1.3.2 Exclusion of oxygen

Resting sGC has a pentacoordinate (5c) heme in ferrous state and is ligated to His105. Many hemoproteins, e.g., hemoglobin, myoglobin, cytoglobin, etc. have a similar histidine-ligated 5c heme and exhibit affinity to NO, CO and O_2_. Despite these similarities, sGC does not bind O_2_ under aerobic conditions [(O_2_) ∼ 260 µM], even under high pressure of pure O_2_ (Martin et al., 2006). The affinity of hemoglobin and myoglobin for O_2_ is enhanced by stabilization of O_2_ ligand *via* the hydrogen bonding (H-bond) to several amino acid residues, especially His63, in the distal side of the heme pocket. The lack of an amino acid residue that can form hydrogen bond to O_2_ was suggested as a possible explanation for such strong exclusion of O_2_ as a gaseous ligand for sGC (Boon et al., 2005). The introduction of such tyrosine residue into the protein fragment containing sGC heme binding domain resulted in some spectral changes that could support the notion of oxygen binding to this heme-containing fragment (Boon et al., 2005). However, when similar mutation was introduced to the full length sGC, no oxygen binding was observed, while the sGC mutant was fully functional (Martin et al., 2006), indicating the exclusion of O_2_-binding in sGC is unlikely due to the lack of distal H-bond-forming residue(s).

#### 1.3.3 Affinity for carbon monoxide

CO is a well-known gaseous ligand for various hemoproteins. *In vivo*, at least 25 hemoproteins were observed to bind CO with K_D_s spanning almost 8 orders of magnitude from low nM to high μM range (Lu et al., 2022). The ability for CO to activate sGC was first discovered in 1987 in a study of the anti-platelet aggregation activity of CO (Brune and Ullrich, 1987). A few years later, experiments with pre-constructed rabbit aorta demonstrated that exposure to ∼100 µM CO results in almost 80% dilation, a vasoactive effect equivalent to the relaxation induced by 100 nM NO (Furchgott and Jothianandan, 1991). In both cases, pure CO gas increased the cGMP content in the studied system by 30%–50%, suggesting that guanylyl cyclase activity may be affected.

Extensive *in vitro* studies demonstrated that cGMP-forming activity of sGC is indeed activated by CO. However, the extent of such activation is very modest, since even with saturated CO sGC is activated only 2–4-fold (Stone and Marletta, 1994; Friebe et al., 1996). Spectral studies clearly demonstrated that binding of CO to the sGC heme results in the formation of a hexacoordinate (6c) carbonyl heme with preserved coordination with His105 (Stone and Marletta, 1994; Kharitonov et al., 1999). Unlike the sGC-NO adduct, the sGC-CO adduct does not undergo any further transformation (Stone and Marletta, 1994). The affinity of sGC for CO has been determined by monitoring the time-resolved UV-Vis spectral changes from the 431 nm absorbance of sGC ferrous heme to the 422 nm peak of sGC-CO at various levels of CO. Soluble GC has low affinity for CO with an estimated K_D_ of 240–260 µM (Kharitonov et al., 1999; Martin et al., 2006) (Table 1). With the exception of toxic exposure to CO gas, the endogenous concentrations of CO are in nanomolar range (Coburn et al., 1963; Coburn, 1973; Marks et al., 2002). At these concentrations of CO it seems unlikely that a substantial sGC-dependent increase of cGMP is possible. The problem of CO-dependent regulation of sGC is further exacerbated by the high affinity for CO of the abundant cellular heme-containing globins. CO binding affinity of hemoglobin, myoglobin, neuroglobin or cytoglobin is 3–6 orders of magnitude higher than that of sGC (Lu et al., 2022). Therefore *in vivo*, it is highly unlikely that any significant amount of sGC-CO is formed.

**TABLE 1:** Ligand specificities of the α_1_β_1_ sGC.

Complex	K_D_ (range)^a^	k_off_ (range) ref	k_on_ (range) ref
NO-sGC complex			
6c sGC-NO	54 nM	27 s^−1^ (Tsai et al., 2011b)	4.5 × 10^8^ M^−1^s^−1^ (Tsai et al., 2011a)
5c sGC-NO_p_	1.25–4.2 pM	6 × 10^−4^ s^−1^ (Kharitonov et al., 1997a)	1.4–4.5 × 10^8^ M^−1^s^−1^ (Zhao et al., 1999; Tsai et al., 2011a; Yoo et al., 2015)
5c sGC-NO_d_	270–850 pM	0.12 s^−1^ (Tsai et al., 2011a)	1.4–4.5 × 10^8^ M^−1^s^−1^ (Zhao et al., 1999; Tsai et al., 2011a)
5c-sGC (GTP)—NO_d_	90–280 pM	0.04 s^−1^ (Kharitonov et al., 1997b)	1.4–4.5 × 10^8^ M^−1^s^−1^ (Zhao et al., 1999; Tsai et al., 2011a)
CO-sGC complex			
6c sGC-CO	260 µM	10.7 s^−1^ (Martin et al., 2006)	4 × 10^4^ M^−1·^s^−1^ (Martin et al., 2006)

^a^ The dissociation constant KD is calculated from the koff/kon ratio based on listed koff and kon values.

It should be noted that the extent of sGC activation by CO is drastically affected by allosteric sGC stimulators. A number of small molecules that sensitize sGC to gaseous ligands have been described over the last 2 decades (Sandner et al., 2019). These compounds by themselves are weak activators of sGC (2–5 fold over basal activity). However, in combination with CO they result in a synergistic activation of sGC’s cGMP-forming activity. E.g., in the presence of the sGC stimulators, such as YC-1 or BAY41-2272, the typical 2–4 fold CO-dependent activation of sGC activity is boosted to a 100 fold activation (Friebe et al., 1996; Martin et al., 2005b). Time-resolved spectroscopic studies demonstrated that in the presence of YC-1 sGC exhibits an increase in CO affinity by an order of magnitude and an almost three orders of magnitude faster CO association constant (Kharitonov et al., 1999). Resonance Raman studies of sGC-CO complex demonstrated that the addition of BAY41-2272 results in a substantial conversion of 6C sGC-CO complex into a 5 sGC-CO complex (Martin et al., 2005b). These data indicate that BAY41-2272 facilitates the cleavage of His105-heme bond, which is known to be a key step in the activation of sGC by NO (see below), and explain well the high cGMP-forming activity of sGC exposed to both CO and sGC stimulators YC-1 and BAY41-2272. Other structurally similar sGC stimulators most likely have similar effect. Some of these stimulators, such as riociguat and vericiguat have been approved for treatment of conditions of pulmonary hypertension and heart failure, respectively (Ghofrani et al., 2013a; Ghofrani et al., 2013b; Armstrong et al., 2020). It remains to be determined whether the improved affinity for CO and elevated cGMP-forming activity of sGC-CO complex is a contributing factor to clinical effects of these sGC targeting stimulators.

#### 1.3.4 The process of sGC interaction with nitric oxide ligand

##### 1.3.4.1 The multistep process of nitric oxide binding-formation of 6c sGC-nitric oxide and conversion to 5c sGC-nitric oxide

Initial mechanistic studies revealed that NO interaction with sGC heme is a two-step process (Makino et al., 1999; Zhao et al., 1999), which can be summarized by the following simplified scheme:

5c His105-heme + NO ↔ 6c His105-heme:NO ↔ His105 + 5c heme:NO.

**FIGURE 2:**
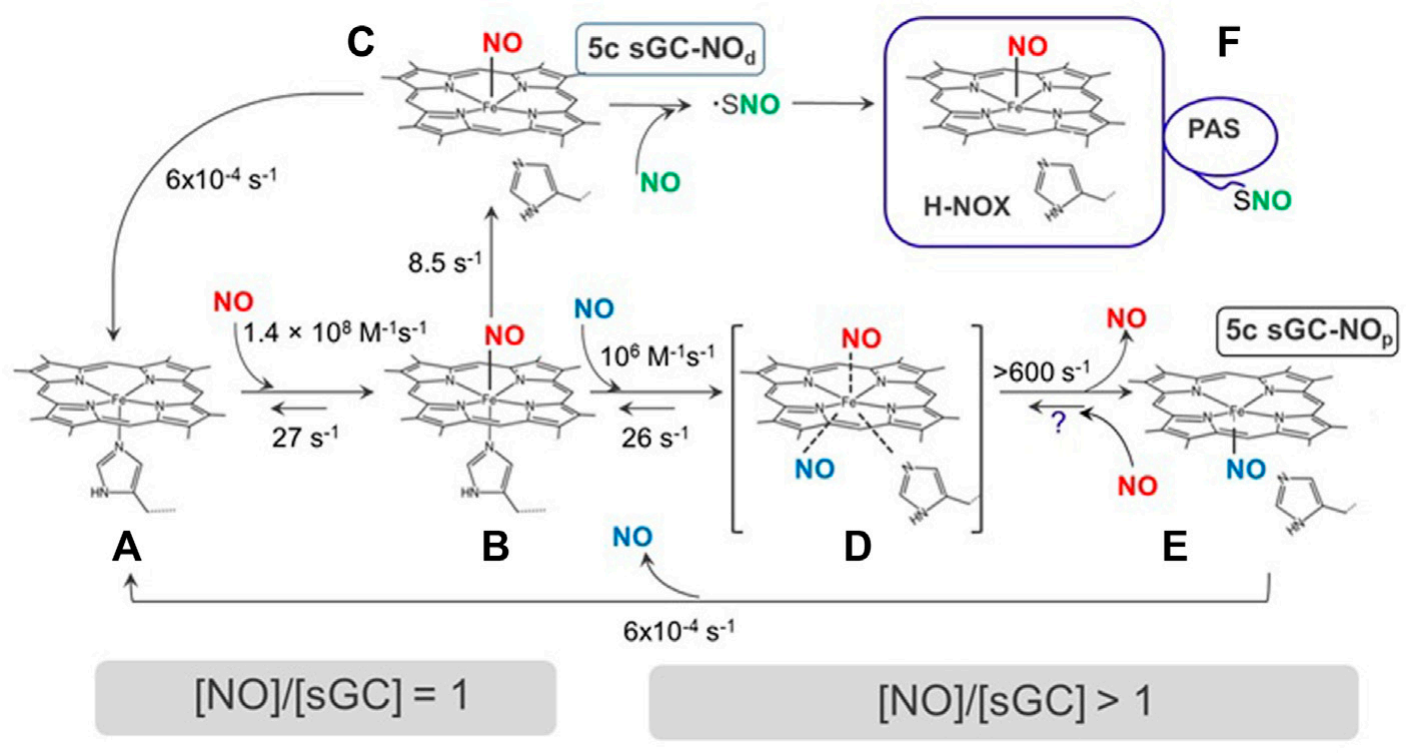
The interactions of NO with sGC. sGC **(A)** reversibly binds to NO to form a 6c sGC-NO complex **(B)**. The 6c sGC-NO complex subsequently converts to a 5c sGC-NO_d_ complex **(C)** irreversibly due to the rupture of Fe-His bond. NO in the 5c sGC-NOd presumably stays on the distal side of heme (denoted by the subscript “d”). In the presence of excess NO, a second NO binds to B to form a transient ternary complex **(D)**, which converts to another 5c sGC-NO_d__p_ complex **(E)** rapidly from both Fe-His bond rupture and dissociation of distal NO. NO in **(E)** presumably stays on the proximal side of the heme (denoted by subscript “p”). Species **(F)** with *S*-nitrosylthiol(s) may form in the excess of NO. Question mark represents an unconfirmed reaction.

**FIGURE 3:**
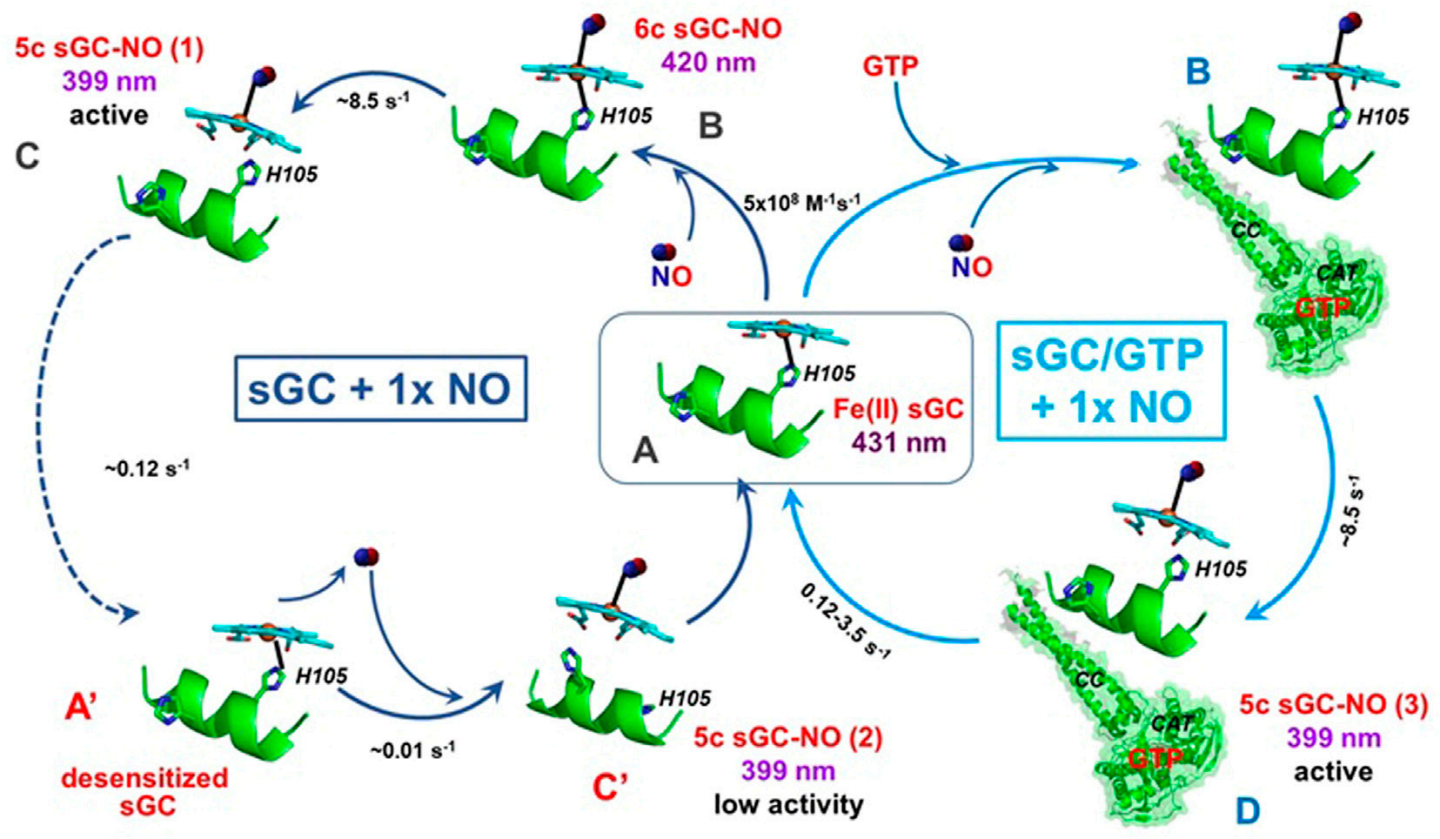
Interactions between sGC with stoichiometric NO. In the absence GTP (blue, left circle), sGC **(A)** binds stoichiometric NO to form 6c sGC-NO complex **(B)**, which converts into the 5c sGC-NO_d_ **(C)** complex. The 5c sGC-NO_d_ is fully active, but converts into a desensitized form of sGC **(A′)**, which binds NO very slowly to form a low activity 5c sGC-NO_d_ complex **(C′)**. In the presence of GTP (cyan, right circle), 5C sGC-NO_d_ does not convert to the desensitized form of sGC, presumably due to the conformational effects induced by GTP. Instead, the active conformation **(D)** is achieved with NO bound to the distal side of the heme. The Soret absorbance of resting sGC is 431 nm while the band shifts to 420 nm and 399 nm in 6c and 5c sGC-NO complexes, respectively.

In the first step, 5c heme-sGC binds NO with an essentially diffusion-limited rate of 1.4 × 10^8^ M^−1^s^−1^ (Zhao et al., 1999; Martin et al., 2005a; Martin et al., 2006) to form a 6c sGC-NO complex (Figures 2A,B), as determined by time-resolved stopped-flow spectroscopic observations, following spectral changes at wavelengths marked in Figure 3. This 6c sGC-NO complex is transient, as it irreversibly converts into a 5c sGC-NO complex after cleavage of the bond between heme iron and His105 (Zhao et al., 1999) at a rate of 8.5 s^−1^. The sequence of events dictates that NO is positioned on the distal side of the heme (designated as “5c sGC-NO_d_” in Figure 2, complex C).

##### 1.3.4.2 Two hypotheses of the second nitric oxide binding

Subsequent studies demonstrated that this two-step NO-binding is more complex and is affected by the amount of available NO. The conversion from the 6c sGC-NO complex to the 5c sGC-NO_d_ complex is due to the strong negative *trans* effect of NO which weakens the Fe-His bond opposite of NO (Hough et al., 2011). Although the conversion from 6c sGC-NO to 5c sGC-NO_d_ complex seems to be a simple intramolecular conversion, early measurements determined that the dynamics of 6c sGC-NO→5c sGC-NO_d_ step depends on NO concentration (Zhao et al., 1999; Martin et al., 2005a), i.e., the conversion is faster at higher concentrations of NO. This NO-concentration dependence data implies the involvement of additional NO molecule. Another line of studies implicating the need for additional NO comes from investigation of sGC activity in the presence of stoichiometric NO. Russwurm and colleagues were the first to demonstrate that sGC exposed to equimolar NO does not exhibit the maximal activity, which can be achieved only after additional NO is provided (Russwurm and Koesling, 2004). Later studies independently confirmed this phenomenon and provided additional information (Cary et al., 2005; Tsai et al., 2011a), which will be discussed in more details later in this review.

Using carefully timed sequential addition of ^14^NO and ^15^NO ligands and subsequent rapid freezing and EPR analysis, it was demonstrated that in the presence excess NO a 5c sGC-NO_p_ complex is formed with NO positioned on the proximal side of the heme (Figure 2, complex E). The 5c sGC-NO_p_ complex forms through the reaction of 6c NO-heme with excess NO with a rate constant of ∼10^6^ M^−1^s^−1^. This presumably occurs *via* a transient NO-heme-His (--NO) quaternary complex, which irreversibly converts at > 600 s^−1^ to 5c NO_p_ (Figure 2, B→D→E) (Martin et al., 2012). The existence of 5c NO_p_ nitrosylated heme was first demonstrated by the X-ray structure of A*lcaligenes xylosoxidans* cytochrome c’ (Lawson et al., 2000). Similar 5c NO_p_ was later observed in the crystal structure of NO-bound H-NOX protein from *Shewanella oneidensis* (*So* H-NOX) (Herzik et al., 2014). Due to the high degree sequence and structural similarity between *So* H-NOX and *ß*_1_ H-NOX domain of sGC H-NOX, 5c NO_p_ likely forms in sGC in the presence of excess NO.

The other proposed mechanism involves protein modification by a second NO molecule that follows the binding of first NO to the heme. A putative thiol-NO adduct in the form of nitrosothiol or thionitroxide has been postulated (Figure 2, state F) (Fernhoff et al., 2009). While the formation of 5c sGC-NO_p_ complex was based on empirical spectroscopic data, the alternative mechanism of thiol-NO adduct formation was postulated based on the biphasic nature of NO-dependent activation of purified sGC. A number of studies reported that when only stoichiometric amount of NO donors is provided, cGMP-forming activity of the resting sGC is activated only ∼10 times (Russwurm and Koesling, 2004). However, in the presence of excess NO a full activation (> 100 fold) was achieved (Russwurm and Koesling, 2004; Cary et al., 2005). Using a thiol reactive probe methyl methanethiosulfonate (MMTS), the authors demonstrated that in MMTS-treated sGC the NO-heme complex was formed, but full activation was not achieved even when excess NO donor was used. Thus, the existence of a thiol-NO adduct needed for full activation was postulated (Figure 2, state F).

The importance of cysteine thiol in sGC activity and function is well recognized (Beuve, 2017). A number of studies demonstrated that several sGC cysteines form nitrosothiols (Sayed et al., 2007; Sayed et al., 2008; Crassous et al., 2012; Beuve et al., 2016). However, all of the detected nitrosothiols were associated with desensitization of sGC and lower cGMP-forming activity. Thus, the exact nature of sGC thiol-NO adduct(s) in the presence of excess NO and the correlation between such thiol-NO adduct(s) with full sGC activation remains to be determined.

While the nature of the second NO binding site remains a matter of dispute, it is highly plausible that these alternative mechanisms are relevant only *in vitro* research, where purified sGC is used and excess NO is provided under experimental control. In this respect, it is important to analyze the physiological ratio of NO and sGC. Direct measurements of NO produced in different cells (Sato et al., 2006; Hall and Garthwaite, 2009) and assessment of NO available for sGC activation suggest that physiological level of NO reaches subnanomolar concentrations (Chen and Popel, 2006). At the same time, it has been estimated that intracellular concentrations of sGC, at least in platelets and cerebellar astrocytes, are in micromolar range (Batchelor et al., 2010). These assessments indicate that under normal physiological conditions the interaction between NO and sGC occurs at substoichiometric ratios, where the amount of NO is not sufficient to saturate the available cellular sGC. Therefore, it is likely that studies performed with stoichiometric NO better reflect the processes of NO-sGC interaction that occur *in vivo*.

##### 1.3.4.3 nitric oxide-soluble guanylyl cyclase interaction under stoichiometric nitric oxide condition

*In vitro* time-resolved stopped-flow studies with purified sGC and stoichiometric NO revealed a number of unique features of the NO-sGC interactions under these conditions (Tsai et al., 2011a). It was reported that the 5c sGC-NO complex resulted from the reaction of purified sGC with stoichiometric NO (Figure 3, complex C) is relatively unstable. Unless sGC is engaged in cGMP catalysis (see below), NO dissociates from the 5c sGC-NO complex (in this case 5c sGC-NO_d_) at a rate of 0.12 s^−1^ (Figure 3, C→A′). In the absence of GTP and under stoichiometric NO, the dissociation of NO from the 5c sGC-NO_d_ converts the enzyme into a desensitized state (Figures 3, state A′), which is still capable of rebinding NO, but with a much slower association rate (∼0.01 s^−1^) than the resting sGC (Figure 3, state A). It should be noted that if GTP substrate is provided before the conversion to the desensitized state (Figure 3, state A′), the 5c sGC-NO_d_ complex, exhibits full activation (Tsai et al., 2011a). Once formed, the secondary 5c sGC-NO complex (Figure 3, complex C′) exhibits a much lower cGMP-forming activity, in line with the definition of a desensitized sGC. Several studies reported that the sGC-NO complex formed with a stoichiometric amount of NO has a much lower cGMP-forming activity in comparison with sGC-NO formed with excess NO (Russwurm and Koesling, 2004; Cary et al., 2005). It was also reported that if sGC reacts with stoichiometric NO in the presence of GTP, then the high cGMP-forming activity is restored (Russwurm and Koesling, 2004). In the presence of GTP, the 5c sGC-NO complex generated under stoichiometric NO (Figure 3, complex D) is more stable than in the absence of GTP, does not form a desensitized form and exhibits maximal NO-dependent activation (Tsai et al., 2011a). These data suggest that *in vivo* depletion of the intracellular GTP pool may contribute to the loss of maximal NO-dependent activation of sGC, leading to its desensitized state as complex C’ (Figure 3). Another pathway leading to the desensitization of sGC was observed in response to repeated exposure to NO donors or nitrovasodilators, such as nitroglycerin. In these cases the mechanism of desensitization was linked to the formation of nitrosothiols (Sayed et al., 2007; Sayed et al., 2008), resulting in sGC with lower enzymatic activity. These data highlight the complexity of hypothetical nitrosylation of sGC thiols in both activation and desensitization.

#### 1.3.5 The affinity of sGC to nitric oxide

Soluble GC is considered a specific NO receptor, which possesses a high affinity for NO ligand. The K_D_ values for NO reported for purified sGC enzyme preparation vary in a wide range from 1.2 pM to 54 nM. The binding of NO to sGC heme is very fast. Using stopped-flow techniques a number of studies recorded time-resolved changes in UV-Vis spectra during the formation of sGC-NO complex. In all these studies the association rate constant k_on_ was predicted in the narrow range of 1.4–4.5 × 10^8^ M^−1^s^−1^ (Zhao et al., 1999; Martin et al., 2005a; Martin et al., 2006; Tsai et al., 2011b; Yoo et al., 2015). The k_off_ of 6c NO-sGC was determined as 27 s^−1^, giving an intrinsic K_D_ of sGC for NO, 54 nM. On the other hand, the k_off_ of 5c NO-sGC complexes (sGC-NO_d_ and sGC-NO_p_) vary significantly, depending on experimental conditions. A very slow dissociation rate constant for 5c NO-sGC complex, 6 × 10^−4^ s^−1^, was determined by Kahritonov and colleagues, who used excess NO to form the complex (Kharitonov et al., 1997a). As described above, under these experimental conditions a second NO molecule was shown to bind to the proximal side of heme forming the 5c sGC-NO_p_ complex (Figure 2, complex E). The dissociation of NO from the 5c sGC-NO_p_ complex may be hampered by steric constrains and the absence of the negative *trans* effect of the Fe-His bond. Thus, the formation of a 5c sGC-NO_p_ complex with NO on the proximal side of the heme (Figure 2, complex E) is the most likely explanation for the slow 6 × 10^−4^ s^−1^ dissociation rate constant, although the possibility that a complex-stabilizing thiol-NO adduct is formed cannot be ruled out. The apparent K_D_ for NO in these conditions was estimated to be in the 1.25–4.2 pM range. We summarize the published k_on_ and k_off_ and the intrinsic and apparent K_D_ of sGC for NO and CO in Table 1.

The experiments performed with stoichiometric NO suggest that the 5c sGC-NO_d_ complex formed in this case (Figure 3, complex C) is less stable and the dissociation of NO from the heme occurs at the apparent rate of 0.12 s^−1^ (Figure 3, C→A) (Tsai et al., 2011a). Thus, under sGC:NO ratio of 1:1 the apparent K_D_ for NO is estimated in the range of 270–850 pM. Since in that study the decomposition rate of 5c sGC-NO_d_ was estimated in the absence of NO scavengers and the rebinding of NO to the heme was unavoidable, the apparent K_D_ value for 5c sGC-NO_d_ listed in Table 1 is overestimated and the real value is probably lower. Studies that use NO scavengers after the 5c sGC-NO_d_ complex is formed are needed to determine the real K_D_ value for sGC under physiologic sGC:NO ratios. Finally, when measurements of NO dissociation in conditions of excess NO were performed in the presence of GTP substrate, a 0.04 s^−1^ dissociation rate was determined (Figure 3), predicting a 90–280 pM K_D_ for NO.

Since sGC functions as an intracellular NO receptor, the issue of sGC affinity for NO should be considered under physiological conditions. As we discussed above, sGC in cells is most likely exposed to substoichiometric amount of NO. The intracellular concentrations of GTP is estimated to be around 100–200 µM (Otero, 1990; Hatakeyama et al., 1992), which is close to the K_M_(GTP) value for purified sGC (Schmidt et al., 2003). This also indicates that most of intracellular sGC is in the GTP bound form. These facts strongly suggest that under physiologic conditions sGC’s affinity for NO is most likely in the sub-nanomolar range (Table 1). Yet, the potential picomolar affinity for NO under certain conditions and high micromolar level of sGC in some cells (Batchelor et al., 2010) may explain why intracellular level of cGMP may change even in response to picomolar fluxes of NO (Batchelor et al., 2010).

#### 1.3.6 Factors contributing to sGC ligand selectivity

Thus, sGC exhibits a dramatic ligand selectivity, which enables sGC to sense minimal NO signal in the presence of enormous amount of O_2_ (Hall and Garthwaite, 2009). The key factors that contribute to such selectivity became better understood since the proposal of a “sliding scale rule” (Tsai et al., 2011b). Due to their different electronic structures, NO, CO and O_2_ bind to a 5c heme with dramatically different affinities. Even the heme model compound protoporphyrin IX 1-methylimidazole [PP(1-MeIm)], the simplest form of five-coordinate heme analogue, exhibits a significant ligand selectivity, demonstrated by K_D_ (CO)/K_D_ (NO) ≈ K_D_ (O_2_)/K_D_ (CO) ratios ≈ 10^3^–10^4^.

**FIGURE 4:**
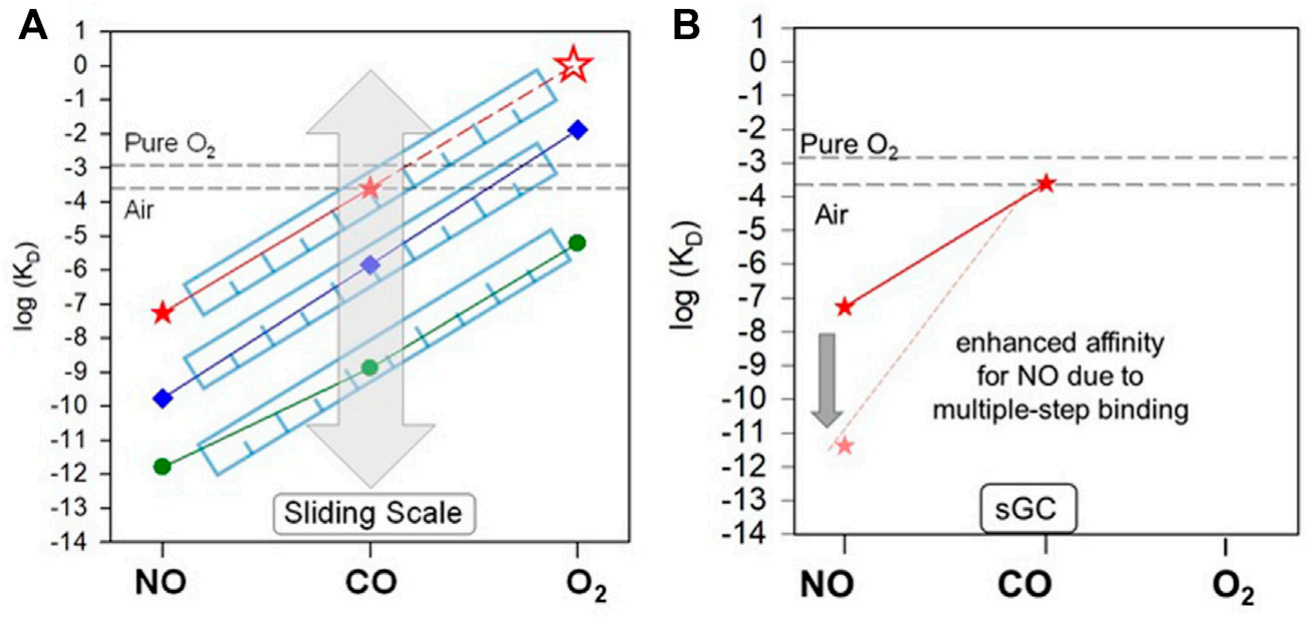
“Sliding scale rule” and sGC ligand selectivity. **(A)** The affinities of sGC for NO, CO, and O_2_ is determined by the proximal stain of heme stemmed from its structural fold. As revealed in the “sliding scale rule”, the structural folds of hemoproteins apply proximal strain through Fe-His bond strength and distal hindrance for the gaseous ligand access to heme (the latter is not applicable to sGC), modulating the affinities of a hemoprotein for NO, CO, and O_2_ simultaneously, but preserving the intrinsic affinity ratios of a 5c heme ligated to a neutral histidine ligand. Preservation of the gaseous ligand affinity ratios to 5c heme and modulation by protein environments in hemoproteins are illustrated by the parallel lines connecting the log K_D_ (NO) to log K_D_ (CO) to log K_D_ (O_2_) of hemoproteins and heme model compounds *versus* ligand type, as shown here by those of sGC (red), H-NOX protein from *Nostoc* sp (*Ns* H-NOX) (blue) and heme model PP(1-MeIm) (green). The “sliding” of parallel log K_D_ (NO)-log K_D_ (CO) -log K_D_ (O_2_) lines is represented by the grey block arrow. The K_D_ (O_2_) of sGC (red empty star) which cannot be determined experimentally is predicted based on the “sliding scale rule” by extrapolating the log KD (NO)-log K_D_ (CO) line of sGC (red dashed line). The [O_2_] in aqueous solutions under 1 atm pure O_2_ and air is represented by the black dashed lines. **(B)** The multiple step NO-binding in sGC, converting the 6c sGC-NO complex to a 5c sGC-NO complex dramatically enhances the affinity of sGC for NO, represented by the block arrow indicating the ∼4 orders of magnitude decrease from K_D_ (NO) to K_D_ (NO)_apparent_.

The large affinity ratios between NO, CO and O_2_ is preserved in hemoproteins with a 5c heme ligated to a neutral histidine proximal ligand, such as sGC. The following discussion focuses on this type of hemoproteins. An extensive bioinformatics analysis of the binding data for all three gaseous ligands from more than a hundred of hemoproteins with a neutral histidine as the proximal ligand, revealed that the K_D_ (CO)/K_D_ (NO) ≈ K_D_ (O_2_)/K_D_ (CO) ≈ 10^3^–10^4^ ratios are preserved despite the significantly different protein folds. Since the affinity ratios between the two ligand pairs are approximately equal, plotting the logarithms of the gaseous ligand affinities [log K_D_ (NO), log K_D_ (CO) and log K_D_ (O_2_)] of a hemoprotein *versus* the ligand type generates an approximately straight line. The lines from different hemoproteins are approximately parallel to each other (Tsai et al., 2011b; Tsai et al., 2012). While the K_D_ (CO)/K_D_ (NO) = K_D_ (O_2_)/K_D_ (CO) ratios are seemingly independent of protein structural folds, the structural fold of a hemoprotein modulates the placement of its log K_D_ (NO)-log K_D_ (CO)-log K_D_ (O_2_) line along the log K_D_ axis (Figure 4). The protein fold adapts the function of the protein to different physiological requirements. A clear application of this pattern, is that it is possible to estimate the affinity of other gaseous ligands in the NO/CO/O_2_ triad, as long as the affinity for one ligand is known (Figure 4). The validity of this approach was experimentally helpful in interpreting the mechanism of the gaseous ligand selectivity of sGC and other heme sensor proteins (Tsai et al., 2012; Wu et al., 2013; Wu et al., 2015; Wu et al., 2017; Wu et al., 2021). Thus, according to the “sliding scale rule”, the K_D_ (O_2_) for sGC is estimated to be ∼1.3 M, a concentration that cannot be achieved in physiologic conditions, considering that oxygen concentration is ∼260 µM in aerobic conditions under normal pressure. As mentioned above, the affinity of sGC for CO (240–260 µM) is outside of any physiological CO concentrations.

A number of strategies used by hemoproteins with neutral histidine to govern gaseous ligand selectivity have been identified by the “sliding scale rule”. The protein structural folds can 1) apply proximal strain to the heme through the proximal histidine ligand; 2) restrict the distal access of a gaseous ligand to the heme; 3) stabilize the oxyferrous heme complex through H-bonds formed between the O_2_ and distal H-bond donor(s); and 4) promote the multiple-step NO binding. As we discussed above, the effect 3) of introduction of a distal H-bond donor residue does not have any impact on sGC affinity for oxygen (Martin et al., 2006), in contrast to the stabilization effect for O_2_ by such distal H-bond donor residues in many hemoproteins.

Among the factors 1) and 2), which universally modify the affinities for all three gaseous ligands in hemoproteins, the proximal strain to the heme plays the most important role in sGC (Tsai et al., 2011b; Tsai et al., 2012). In hemoproteins, the proximal strain can be considered as a “pull” imposed on the heme through the proximal histidine ligand by protein structural folds. Such a “pull” governs the length and strength of the Fe-His bond between the heme iron and the proximal histidine ligand (Jain and Chan, 2003). The strength of the Fe-His bond regulates the bimolecular association rate (*k*_on_) and unimolecular dissociation rate (*k*_off_) of a gaseous ligand, ultimately affecting the affinity of the hemoprotein for the gaseous ligand (Tsai et al., 2012). E.g., sGC has a weaker and longer Fe-His bond than its structural analogues, bacterial H-NOX proteins (Pellicena et al., 2004; Ma et al., 2007; Erbil et al., 2009; Kang et al., 2019). The longer Fe-His presumably generates a strong proximal strain in sGC, leading to the affinities of sGC for all three gases two-order lower than those in bacterial H-NOXs (Wu et al., 2017). Actually, according to the cryo-EM structure of sGC (Kang et al., 2019), the Fe-H105 bond in sGC measures 3.6 Å, the longest among all the hemoproteins known so far. The notion of a weak Fe-His bond in sGC is also supported by its low Fe-His stretching frequency of 204 cm^−1^ (Schelvis et al., 1998) and the very high midpoint redox potential (E_M_) of 187–234 mV compared to other hemoproteins (Fritz et al., 2011; Makino et al., 2011).

**FIGURE 5:**
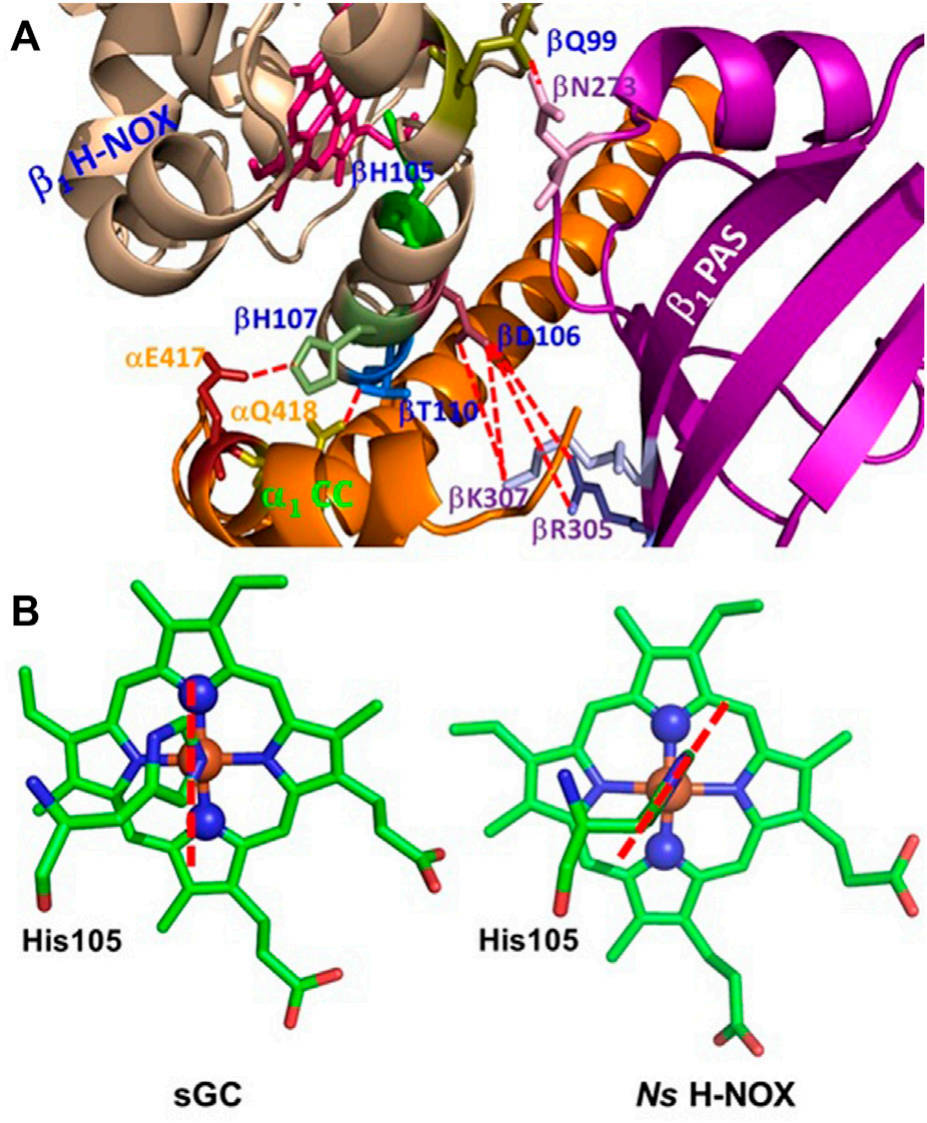
Structural elements which may determine the proximal strain in sGC. **(A)** Possible H-bonding or polar interactions between the residues on or near helix F in *ß*_1_ H-NOX domain and residues from other *α*_1_ and *ß*_1_ domains, represented using red dashed lines. The proximal ligand to the heme, His105, is located on helix F, and these H-bonding or polar interactions may modulate the length and strength of Fe-His105 bond, therefore contributing to the proximal stain of heme. **(B)** In sGC, His105 imidazole ring orients approximately along the line connecting the nitrogen atoms of pyrrole rings B and D of the heme, adopting an “eclipsed” conformation (PDB 6JT0). On the other hand, in *Ns* H-NOX, a bacterial analogue of sGC, the imidazole ring of the proximal ligand His105 is in a “staggered” conformation, orientated more along the line connecting *β* and *δ* methine carbons of the heme (PDB 2Y10). The heme with a staggered proximal histidine ligand usually has stronger affinities for the gaseous ligands.

The strong proximal strain of heme in sGC may come from extensive hydrophobic and polar interactions between the different domains in the *ß*_1_ subunit and between *α*_1_ and *ß*_1_ subunits. Hydrophobic interactions are found between the residues in *ß*_1_ H-NOX and the residues in α_1_ PAS and α_1_ CC domains (Kang et al., 2019). Moreover, charged and polar interactions exist extensively among the residues in *ß*_1_ H-NOX and the residues in *α*_1_ PAS and CC domains and *ß*_1_ CC and PAS domains (Figure 5A) (Kang et al., 2019). These interactions, particularly those involving the residues on *ß*_1_ helix F where His105 the proximal ligand to the heme is located, may modulate the proximal strain of heme and collectively determine the affinity of sGC for the gaseous ligands.

The structural fold of a hemoprotein also restricts the orientation of the projection of the histidine imidazole ring onto the heme protoporphyrin ring plane. This orientation fine-tunes the proximal strain of heme. In hemoproteins, the projection of the histidine imidazole ring onto the heme protoporphyrin ring is either in an “eclipsed” orientation, approximately parallel with the line connecting the nitrogen atoms of two opposite heme pyrroles or in a “staggered” orientation, roughly parallel with the line connecting two opposite methine carbons of the heme (Olson and Phillips, 1997; Tsai et al., 2012). It is found that the “staggered” conformation is relaxed while the “eclipsed” conformation is more constrained, leading to stronger proximal strain and therefore lower affinities for the gaseous ligands (Carver et al., 1992; Quillin et al., 1995; Hargrove et al., 1997; Olson and Phillips, 1997; Andrew et al., 2002; Draghi et al., 2002; Kundu et al., 2002; Kundu and Hargrove, 2003; Hough et al., 2011; Garton et al., 2012). The cryo-EM structure of sGC revealed that the imidazole ring of proximal His105 is in an “eclipsed” conformation (Figure 5B), enhancing the proximal strain in sGC (Tsai et al., 2011b).

While the proximal heme strain diminishes the affinities of sGC for all three gases and allows sGC to effectively discriminate against CO and O_2_, it also seems to be incompatible with sGC’s role as NO receptor. The 54 nM affinity for NO determined for the formation of the 6cNO-sGC heme complex does not seem to fit the role of highly sensitive NO receptor typically ascribed to sGC. In fact many other histidine ligated hemoproteins have higher affinities for NO than sGC (Tsai et al., 2012). However, *in vivo*, sGC may outcompete the other hemoproteins in “seizing” NO due to its multiple-step NO-binding mechanism [effect 4)] (Figure 4B). The strong proximal strain in sGC augments the strong negative *trans* effect of NO which weakens the Fe-His bond opposite of NO (Hough et al., 2011), promoting the conversion of 6c sGC-NO complex to 5c sGC-NO_d_ complex (Figures 2, 3). This 6c to 5c conversion step leads to greatly diminished NO dissociation from sGC, boosting its apparent affinity for NO by at least three orders of magnitude (Figure 4B; Table 1). Even more importantly, sGC has the fastest association rate for NO among known proteins (k_on_ = 4.5 × 10^8^ M^−1^s^−1^), which together with the extremely slow NO dissociation from 5c sGC-NO_d_, kinetically gives sGC a upper hand in NO-binding *in vivo*.

With sGC K_D_ (NO) at sub-nanomolar range, one may wonder how it is possible that NO concentrations as low as 3 pM result in measurable increase of intracellular cGMP (Batchelor et al., 2010). The measurement of NO released by stimulated endothelial or neuronal cells suggests that 40–100 pM NO may exist just outside of these cells (Sato et al., 2006). This value correlates well with the 20–100 pM NO that has been estimated to be released to vascular smooth muscle cells by the endothelium in blood vessels (Chen and Popel, 2006). Both these values are lower than the 90–280 pM K_D_ (NO) calculated for sGC/GTP and the 270–850 pM K_D_ (NO) calculated for sGC (Table 1) in the absence of excess NO. It appears that these NO concentrations are not sufficient to promote the physiological function of sGC. However, calculations of sGC content in platelets or cerebellar astrocytes predict cellular sGC concentration of 2 µM, which is significantly higher than the available NO. In case of such a tremendous excess of sGC (>10^4^- fold), NO binding conditions are far from equilibrium, and the contribution of the very fast NO-sGC association rate far outweighs any dissociation of NO. Due to the very fast NO-sGC association rate sGC effectively competes with other potential NO-binding hemoproteins. Under such conditions, sGC acts as a powerful sink for all available NO, even though only a small portion of sGC is activated. Due to a high turnover number of at least 3,000/min, even a minor fraction of high cGMP output state of NO-activated sGC is sufficient to generate a 10–60 nM/s flux of cGMP sufficient to initiate the downstream signal cascade mediated by cGMP-dependent kinases (Francis and Corbin, 1994; Vaandrager et al., 2005).

## 2 Conlusion

Soluble GC uniquely evolved to sense low non-toxic levels of NO and respond to elevated NO levels by increasing its catalytic ability to generate cGMP and promote downstream signaling. sGC exhibits remarkable selectivity for gaseous ligands, demonstrating no binding of oxygen, affinity for CO at non-physiologic concentration and sub-nanomolar affinity for NO. This selectivity is based on the intrinsic selectivity of the neutral histidine-ligated heme, which is substantially modulated by a strong proximal strain exerted by sGC structural folds on the Fe-His bond. This proximal strain diminishes the affinities of sGC for CO and O_2_, and NO. On the other hand, the affinity of sGC for NO is tremendously enhanced through multistep NO-binding mechanism, resulting in a sub-nanomolar K_D_ (NO). This sub-nanomolar affinity for NO, together with the fastest NO association rate and a significant sGC excess over the amount of NO generated under physiologic conditions provides sufficient cGMP-generating activity to promote and sustain the downstream cGMP-dependent signaling.
